# 823 12-Year Look into Acute Ocular Burn Management at a Single Institution

**DOI:** 10.1093/jbcr/iraf019.354

**Published:** 2025-04-01

**Authors:** Hilary Liu, Benjamin Scott, José Arellano, Christopher Fedor, Mare Kaulakis, Garth Elias, Alain Corcos, Jenny Ziembicki, Francesco Egro

**Affiliations:** University of Pittsburgh Medical Center; University of Pittsburgh Medical Center; University of Pittsburgh Medical Center; University of Pittsburgh School of Medicine; University of Pittsburgh School of Medicine; University of Pittsburgh Medical Center; University of Pittsburgh Medical Center; University of Pittsburgh Medical Center; University of Pittsburgh Medical Center

## Abstract

**Introduction:**

Ocular burns, often resulting from workplace accidents, chemical exposures, or thermal incidents, can lead to severe vision impairment or blindness. Management of ocular burns varies, lacking a definitive treatment algorithm. This study reviews the management and complication rates of ocular burns at a single institution.

**Methods:**

A retrospective cohort study analyzed patients with ocular burns treated at an ABA-verified burn center from January 2012 to July 2023. Data included demographics, injury characteristics, treatments, operations, and complications.

**Results:**

Over a 12-year period, 50 patients with 81 ocular burns were treated. The cohort was 84% male, with a mean age of 39.4±20.4 years and mean BMI of 26.1±9.4. Among these patients, 32% (n=16) were smokers. Thermal injuries accounted for most burn etiologies (78%; n=39), followed by chemical (20%; n=10) and electrical (2%; n=1). The average total body surface area (TBSA) affected was 19.9±22.5%.

Corneal injuries, primarily abrasions, occurred in 50% (n=25) of cases, and 12% (n=6) involved the eyelids. The most common immediate treatment was eye irrigation (36%; n=18), especially for chemical burns. 84% (n=42) of patients received erythromycin ointment, 40% (n=20) antibiotic eye drops, 24% (n=12) mydriatic eye drops, 18% (n=9) prednisolone eye drops, and 12% (n=6) ketorolac eye drops.

Complications included two cases of vision loss. One patient required extensive surgical interventions, including a corneal transplant. Another developed acute traumatic cataracts and underwent cataract surgery. One case of entropion required two surgeries. There were two deaths due to burn-related complications.

**Conclusions:**

Chemical burns represent a significant portion of ocular burn cases, with irrigation serving as a primary treatment. While antibiotic therapy is generally effective, severe burns may necessitate future ophthalmic or oculoplastic procedures due to potential complications like eyelid contractures.

**Applicability of Research to Practice:**

This study underscores the importance of timely irrigation for chemical burns and the use of antibiotic therapy in managing ocular burns. It highlights the need for standardized protocols and early intervention to prevent complications like vision loss and the need for surgeries, offering valuable guidance for improving patient outcomes.

**Funding for the Study:**

N/A

